# Sociolinguistic Context Constrains Bilingual Advantages in Metacognition

**DOI:** 10.1111/nyas.70350

**Published:** 2026-07-22

**Authors:** Leona Polyanskaya, Mikhail Ordin

**Affiliations:** ^1^ Centro de Investigación Micaela Portilla Euskal Herriko Unibertsitatea Vitoria‐Gasteiz Spain; ^2^ Interdisciplinary Centre for European Studies Europa‐Universität Flensburg Flensburg Germany; ^3^ Faculdade de Medicina Universidade de Coimbra Coimbra Portugal

**Keywords:** bilingualism, decision‐making, heritage language, metacognition, metacognitive efficiency, minority language

## Abstract

Previous studies have reported enhanced metacognitive efficiency in bilinguals, particularly when both languages are institutionally supported and used across educational and public domains. Whether this advantage extends to heritage‐language bilingualism remains unclear. We tested local and global metacognition in German monolinguals, German–Turkish bilinguals, and German–Slavic bilinguals using three language‐learning tasks targeting semantic inference, statistical learning, and artificial grammar learning. Contrary to predictions based on bilingual advantage and typological‐distance accounts, heritage bilinguals did not show enhanced local metacognitive efficiency. German–Turkish bilinguals showed lower local metacognitive efficiency than monolinguals and German–Slavic bilinguals across tasks, and German–Slavic bilinguals showed no consistent advantage over monolinguals. By contrast, group‐level measures suggested stronger global metacognitive calibration in bilingual participants. These findings indicate that bilingualism does not uniformly enhance metacognition and that sociolinguistic factors, including language status, educational use, and domain breadth of language use, may constrain or override effects of typological distance. The results distinguish local confidence monitoring from broader self‐performance evaluation and identify heritage‐language bilingualism as an important boundary condition for claims about bilingual metacognitive advantage.

## Graphical abstract

Earlier research showed enhanced metacognitive efficiency in bilinguals in language tasks (activities that rely on the cognitive machinery employed for language processing). We investigated whether this advantage extends to heritage language speakers. We did not observe better local metacognition in heritage language speakers. Global metacognition, by contrast, is enhanced in bilinguals despite sociolinguistic context constraints imposed by restricted use of heritage languages in society, in which a different dominant language is spoken.

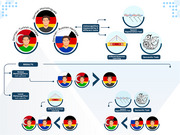

## 1 Introduction

Metacognition is a faculty that allows monitoring one's own mental states and cognitive processes, tracking accuracy of own decisions, consequences of past decisions, and estimating uncertainty in the environment that may affect the consequences of future decisions [1, 2, 3, 4]. Efficient monitoring processes pertaining to metacognition allow for adapting decisions and actions to new environments and circumstances, controlling future behavior [5, 6, 7, 8, 9, 10, 11].

Metacognition operates on different timescales. Trial‐by‐trial evaluation of one's cognitive performance and estimation of uncertainty and error likelihood at the moment of decision‐making is referred to as local metacognition. Global metacognition, by contrast, operates on a longer timescale and refers to overall evaluation of one's success in a particular activity or domain. Local metacognition allows adjusting confidence in one's decision; it is immediate and task‐specific. Although global metacognition is integrative over multiple trials and sessions, it serves to calibrate beliefs about one's general competence [12, 13, 14, 15]. Local metacognition tracks performance in real time, while global metacognition shapes enduring self‐concepts and expectations [16].

Local metacognition may be tapped on by asking an individual how confident (s)he is in a particular decision or how well he has performed in a certain task [17, 18, 19]. Better metacognition is associated with a higher sensitivity to situations in which the error likelihood is increased, which leads to adjusting the confidence level accordingly. High metacognitive sensitivity results in lower confidence assigned to decisions that are more likely to be wrong or in situations in which the evidence for making the forced decision is inconclusive [10, 20, 21, 22]. This may also lead to paradoxical situations that have important societal implications: individuals who are better able to identify erroneous decisions adapt certainty in their own judgments and, hence, express their opinions with lower confidence [23, 24, 25]. The human audience, however, tends to assign higher weight to statements that are expressed with higher confidence [26], which is also true of important situations such as listening to witnesses in court or discussing alternatives in a panel of advisors. If the probability of an error is low, such a strategy may be beneficial because it saves time, but in the context of high uncertainty, assigning weights to speakers on the basis of their level of confidence may entail undesirable consequences and increase the risk of incorrect decisions at the collective level. Global metacognition shapes general self‐evaluations, affects resilience and recovery from failures, and influences major life decisions [15, 27]. Its overarching function is to guide the selection of tasks or domains in which individuals expect to be most effective. Given important societal implications [23, 24, 25, 26, 27], we need to understand the factors that may influence metacognition.

Earlier studies have shown that local metacognition at the individual level may be modulated by a variety of factors, including general intelligence (IQ), individual cognitive style (e.g., the tendency to make decisions on the basis of analytical thinking vs. understanding others’ emotions and intentions), cognitive flexibility (selective and deliberate switching between tasks or different sets of rules), educational level, political affiliation, and life history [6, 24, 28, 29, 30, 31, 32, 33, 34, 35, 36]. At the group level, metacognitive differences between populations may emerge due to cultural differences or linguistic environments. Bilinguals, for example, are known to outperform monolinguals in metacognition [37]. Mastering several linguistic systems involves many cognitive processes related to phonological memory [38] and attention [39], raising metalinguistic awareness or conscious awareness of multiple systems, and enhancing the ability to evaluate the sense of cognitive performance.

However, bilinguals do not constitute a homogeneous population [40]. Bilinguals differ in multiple dimensions, including age of acquisition (AoA), relative proficiency in their languages (whether they are more proficient in one of their languages or their proficiency in both languages is balanced), bilingual literacy (whether they are literate in both languages or have written skills in only one of their languages), and whether they use both languages across all operational domains or keep their languages apart to different situational contexts (one language for home use and the other language for professional and educational settings). The specific aspects of bilingualism that contribute to metacognitive advantage remain unclear.

Polyanskaya et al. [41] added another aspect: typological differences between bilinguals’ languages. They argued that the need to monitor different processing strategies required for the production and comprehension of structurally different languages provides natural training for self‐monitoring of one's own performance and of the environment, leading to enhanced metacognitive sensitivity in language tasks. This hypothesis was tested on a statistical artificial language learning task with Basque–Spanish (typologically different languages) and Catalan–Spanish (typologically similar languages) bilinguals, who were compared with Castilian Spanish monolinguals.

The Basque and Catalan languages in Spain have the status of official languages that are used across all communicative domains. They are obligatory media of education at school, not only taught as a subject but also used as a medium for other subjects (universities also provide an opportunity to receive a degree in a regional language). An officially confirmed B2 level in Basque is a minimum requirement to receive a certificate of secondary education and a prerequisite for most public servant jobs. Regional language is used in administrative bodies, and all official documents are issued in two languages. However, this type of bilingualism in the contemporary world is more an exception than a norm.

In this study, we a tested the hypothesis of Polyanskaya et al. [41] on a wider range of linguistic environments and language tasks that engage the same cognitive machinery used when natural speech is processed. We used inferential word learning [42, 43, 44] and artificial grammar learning [45, 46], and a statistical learning task [47, 48, 49, 50], and bilingual advantage was reported only for local metacognition. We tested the hypothesis with cohorts of bilinguals in Germany who use either Turkic languages (Turkish) or Slavic languages (Russian, Polish, and Czech) in addition to their ambient language (German). Turkish and Russian are heritage languages that do not have a status of official languages. Heritage languages are naturalistically acquired and spoken at home or in close communities, in a limited range of social contexts, in a society, where another language is dominant [51, 52]. Usually, competence of heritage speakers differs from that of monolingual peers from the environment, in which the same language is dominant [53].

Morphologically and syntactically, Germanic and Slavic languages share more structural features than do Germanic and Turkic languages. Turkish is a left‐branching language with an SOV word order and an agglutinative morphological system; Germanic and Slavic languages are right‐branching with an SVO word order and fusional morphology. With respect to prosody, German and Russian (or Polish/Czech) are also more similar to each other than German and Turkish. Strict phonotactic rules in Turkish result in a predominance of simple *consonant–vowel–optional consonant* syllables without complex consonantal clusters. Predictable syllable structure, obligatory vowel harmony, and weak vowel reduction are associated with low durational variability of the syllables, yielding a “syllable‐timed” or regular rhythm, which perceptually emerges from a roughly equal duration of vowels and syllables. German and all Slavic languages, on the other hand, are associated with weak phonotactic rules allowing complex consonantal clusters, irregular rhythms that perceptually emerge from the high durational variability of syllables and vowels. Durational variability is further increased by the phonological opposition of long and short vowels in German, and by strong reduction in unstressed vowels. Rhythmic patterns determine segmentation strategies that are more useful for detecting the edges of lexical and phrasal components in typologically different languages by employing different weightings to speech constituents (morae, syllables, feet, and interstress intervals) [54, 55, 56]. The unmarked position of lexical stress in Turkish is aligned with the word‐final syllable. By contrast, in German and Slavic languages, lexical stress is aligned with the word‐initial syllable. Such differences require different processing strategies [57, 58, 59]. On the basis of the hypothesis of Polyanskaya et al. [41], when controlling for IQ, relative language proficiency and bilingual literacy, German–Turkic bilinguals should have more enhanced metacognitive abilities compared to German–Slavic bilinguals because the latter pair is typologically more similar than the former pair is, and both languages in the latter pair can be processed by applying the same cognitive strategy.

Although metacognition is often considered a domain‐general faculty [60, 61], understanding the reliability and fallibility of one's own cognitions also depends on one's experience with a given task or environment. Experience with the environment is necessary to estimate the consequences of wrong decisions and hence to reflect on the soundness of one's own cognitions [62]. Immersion in a bilingual environment is expected to have the most pronounced effect on metacognition in the verbal domain. Selective attention to structurally different systems allows bilinguals to use their experience with multiple inputs and different systems of cues that always need to be weighted, subject to the current state of the linguistic environment [63, 64, 65]. Thus, the linguistic experience of bilinguals can lead to metacognitive enhancement in language tasks, which can potentially spill over beyond the language domain.

In addition to testing the hypothesis of Polyanskaya et al. [41] on a wider range of populations and tasks, this study had a more applied aim. Language used as a medium of education in bilingual environments can also play a major role in modulating bilingual advantages in metacognition. Ordin and Polyanskaya [66] compared metacognition in language tasks in Danish–German bilinguals who either received or did not receive education in their minority language (Danish in Germany and German in Denmark). The study showed that adding a minority language as a medium of education (i.e., as the language of instruction, not as a subject) enhanced bilingual metacognition, and this enhancement could not be attributed to purely structural language properties because bilinguals in both groups had the same languages (Danish and German) in their inventory and did not differ in IQ, demographic factors, or language proficiency. Therefore, the observed metacognitive advantage was accounted for by language use in educational settings.

Metacognition is known not only to monitor but also to regulate behavior by adapting behavioral responses to changes in the environment when a decision‐making or learning strategy becomes suboptimal [1, 3, 4, 67, 68, 69, 70, 71]. This metacognitive control has important bearings on academic success [6, 72, 73, 74]. Hence, it is important to know whether a bilingual metacognitive advantage emerges when the minority or heritage language is not used in educational settings.

We measured local metacognition as a trial‐by‐trial fluctuation of confidence, with the assumption that better metacognition is associated with better tracking of accuracy with confidence ratings (given high metacognitive skills, trials when the error is more likely attract lower confidence, which leads to lower average confidence in the wrong responses than in the correct responses). This measure of local metacognition is accompanied by a measure of global metacognition when participants need to evaluate their general performance on each task (not on separate trials). Local and global metacognition are related faculties [14], although in certain circumstances, these faculties can be disassociated [75] because they rely on different neural and cognitive mechanisms and therefore need to be measured separately.

In particular, the objectives of our study were (1) test the hypothesis that typological distance between languages in bilinguals’ inventory enhances metacognition in all language tasks, which engage the cognitive mechanisms that are brought to bear by processing language systems; and (2) estimate whether bilingualism is associated with local (trial‐by‐trial fluctuations in confidence in one's decisions) or global (general evaluation of self‐performance in a particular task or domain) metacognition.

## 2 Methods

### 2.1 Experiments

We measured metacognition in three language tasks, namely, semantic (inferential word learning), syntactic (artificial grammar learning), and general statistical learning related to language abilities [50, 76, 77]. The details of the tasks and material are presented in Ordin and Polyanskaya [66]; below, we briefly describe them.

### 2.2 Inferential Word Learning

We generated 20 pseudowords using UniPseudo (a universal pseudoword generator, New et al. [78], and the Lexique lexical database [79], following the regularities of German phonotactics (http://www.lexique.org/shiny/unipseudo/). The list of pseudowords and the details of the algorithm are in Ordin and Polyanskaya [66]. During the experiment, the participants saw two images that differed by a minor detail (e.g., one image depicted a table and the other image the same table with a flower on it), and the task was to select a table with a “dinf.” Through inference, people selected a table with a flower and linked a pseudoword with (dinf) the object. In the following trial, they could see a girl with a flower and a girl with a spider, and when asked to choose a girl with a “höso,” they chose a girl with a spider and associated a new pseudoword for spider. After 20 words were introduced, we administered a test involving seeing two images (e.g., a flower and a spider) and choosing “dinf” and then indicating on a 4‐point scale a confidence level for the choice.

### 2.3 Statistical Learning

For a statistical learning experiment, we used a conventional Saffran‐style paradigm with six nonsense tri‐syllabic triplets: KI‐TE‐PO; MA‐LE‐RU; PU‐RI‐ME; KE‐PA‐TI; RA‐LO‐KU; MO‐LU‐TO. The triplets were concatenated one after another into a continuous acoustic sequence so that no one occurred twice consecutively (each triple was embedded 80 times). The participants were told that they were going to listen to an alien language and that they had to detect and memorize the words of this language. Upon listening to the familiarization stream, they performed the test. During the test, they heard two triplets, one taken from the acoustic familiarization stream and the other from a foil composed of the same syllables in the same order, in which these syllables never occurred consecutively in the familiarization stream. The participants had to choose whether the first or the second triplet in the pair was a word from the alien language and then indicate their level of certainty on a four‐point scale. In total, there were 12 trials (each triplet in the test occurred in the first and second positions in the test pair).

### 2.4 Artificial Grammar Learning

**FIGURE 1:**
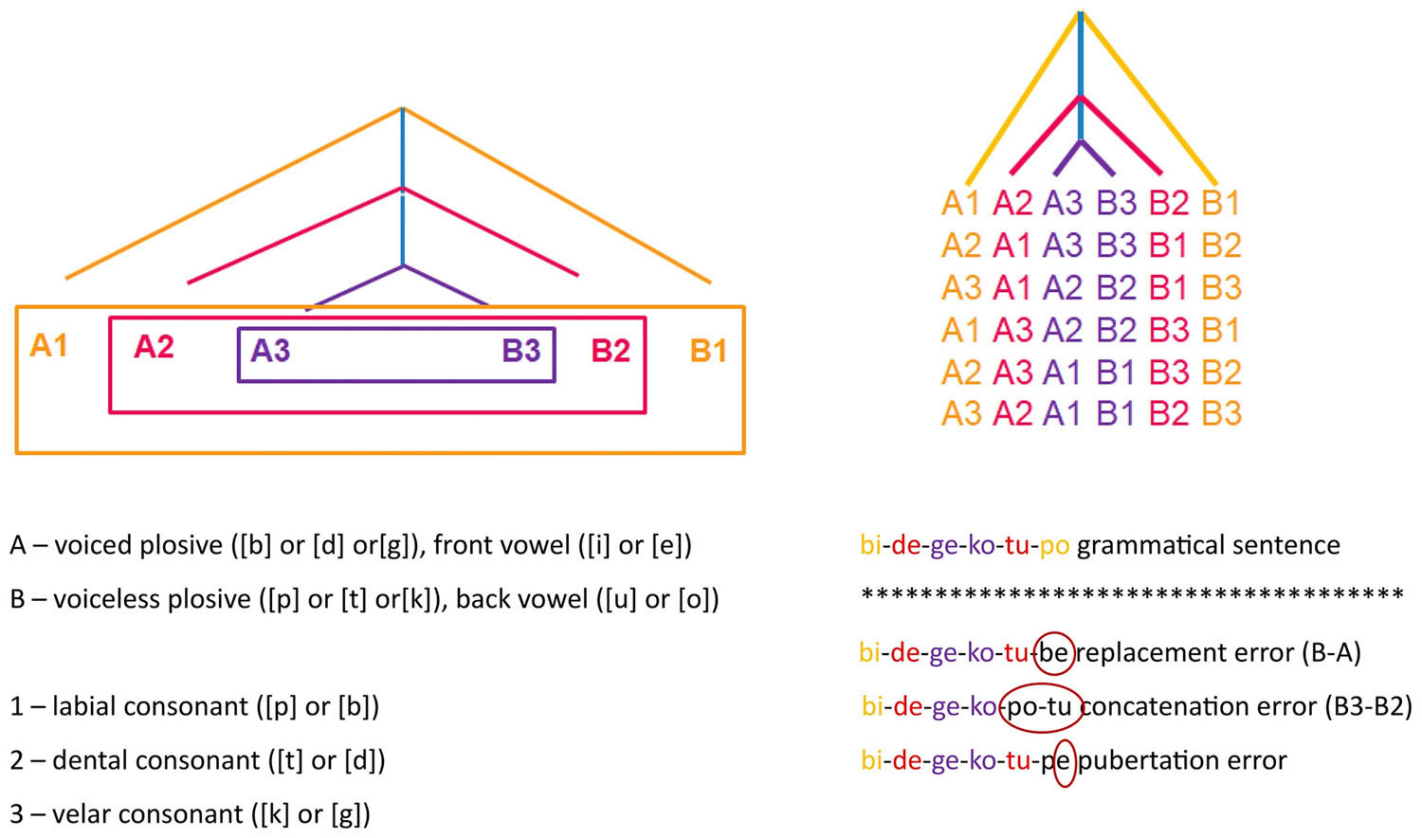
Artificial grammar representation. Possible subunits A1B1, A2B2, and A3B3 are embedded into each other. Syllable A is manifested by a consonant‐vowel syllable combination of a voiced plosive and a front vowel. Syllable B is manifested by consonant‐vowel combination of a voiceless plosive and a back vowel. Numbers 1, 2, and 3 stand for the type of the plosive—labial, dental, or velar, respectively. The tree of potential combinations of how units can be arranged is on the right panel. Possible types of error sequences used in the recognition test are given below the tree.

Participants were informed that they would listen to an alien language and that they had to try to detect the rules. We created a hierarchical grammar with embedded components implemented in six syllable sequences (aka, sentences) (see Figure 1). For example, the unit A1A2 can be embedded into the unit B1B2 as B1A1A2B2, similar to the sentence “The cat, that the dog chased, ran away” (i.e., the unit *the dog cased* is embedded into the unit *the cat ran away*). Subunits A and B differed in voicing, and the positional order (A1, A2, and A3) differed in the place or articulation and vowel fronting. Thus, speech properties were used to create a hierarchical structure that is typical of recursive hierarchical syntactic structures of natural languages. The six‐syllabic sequences (separated by pauses) were concatenated into an acoustic stream that participants listened to, after which the test was administered. The participants listened to a six‐syllable structure and reported whether it was either grammatically correct or wrong in the alien language, and on each trial, we generated a confidence report, also on a four‐point scale. In total, there were 24 trials (with 12 correct sequences and 12 sequences that violated the regularities implemented in the artificial grammar).

### 2.5 Rapid Picture Naming Task

We used 65 images of common objects (e.g., animals, body parts, and household objects) with noncognates in German, Russian, and Turkish. The pictures were shown to the participants one by one, who were then asked to name the object as quickly as possible first in one language and then in the other language of the participants (without mixing the languages). For each correctly named object, a participant received one point, and the difference between the total points collected for two languages was used as a proxy measure of the difference in proficiency between two languages in the bilingual inventory (∆Prof) and which reflects the ease of lexical access [80]. The closer ∆Prof is to zero, the more balanced a bilingual speaker is in terms of relative proficiency; conversely, a higher ∆Prof score reflects skewness in language proficiency. *Proficiency* is a complex construct that also includes fluency in reading/listening comprehension, language use, written, and spoken production. *Lexical access*, or the relative ease of access to languages in bilingual inventories, can measure *relative* proficiency. Lexical access assessed via the rapid picture naming test has been validated as a measure of relative proficiency in bilinguals [80, 81, 82, 83, 84], which we used in his study.

### 2.6 Kaufman Brief Intelligence Test

We used a Kaufman brief intelligence test [85] to measure IQ (we assessed only logical IQ on matrices) because it has been shown to yield replicable and valid assessments of IQ in clinical and research applications [86].

### 2.7 Measuring Metacognition

We operationalized metacognition as the ability to track accuracy with confidence ratings. “Good” metacognition, which allows tracking one's own mental states and cognitive performance depending on environmental uncertainty (also known as “ambiguity in the sensory input”) while considering the outcomes of the past decision, results in detection of trials when the error is more likely, hence adapting (lowering) confidence on such trials [10, 20, 21]. The sensitivity to potential errors leads to higher confidence ratings averaged across all correct responses, than across all wrong responses. Importantly, this does not mean that all the responses with lower confidence are wrong; it may happen that the participant detects an elevated likelihood of an error on a particular trial; but in the end, the participant still gives a correct answer to which a low confidence is attached. However, after averaging, correct responses align with higher confidence ratings compared to wrong responses, given high metacognitive sensitivity of the former.

To estimate the metacognitive sensitivity measures, we used a signal detection approach [18, 19, 87, 88] based on the conceptualization of correct answers with high confidence considered as meta‐hits, correct answers with low confidence as meta‐misses, wrong answers with high confidence as meta‐false alarms, and wrong answers with low confidence as meta‐correct rejections. As this confidence scale is not binary, it is possible to build the meta‐ROC curve for each individual by estimating a pseudo‐*d*′ that optimally fits confidence ratings (i.e., a would‐be *d*′, if the given confidence ratings optimally discriminated between correct and wrong responses). We refer to such an estimated would‐be *d*′ as type‐2 *d*′, or meta‐*d*′ (type‐1 *d*′ is the conventional *d*′ calculated on accuracy responses reflecting cognitive performance).

To fit accuracy data (type‐1 or cognitive performance) to the observed confidence data (type‐2 or metacognitive performance), we used a Bayesian modeling approach [89]. We used the hierarchical Bayesian estimation method proposed in Fleming [90] and adopted it for the design of our experiments. Given that meta‐*d*′ (metacognitive sensitivity) is reported to scale with cognitive performance, we used the *M*‐ratio (meta‐*d*′/*d*′), a measure known as metacognitive efficiency or metacognitive sensitivity given an individual level of cognitive performance.

### 2.8 Procedure

All the participants had to come to the experiment twice, on different days. On the first day, we explained the procedure, obtained signed informed consent, and conducted an interview (to ensure that the participants met the inclusion criteria of bilingualism, namely, that they were exposed to and acquired both languages naturally—not through formal education—from birth). We also asked whether they were born in Germany and, if not, at what age they moved to Germany; whether they were religious and regularly followed religious practices and attended church/mosque; and whether they were literate in both languages or had only oral proficiency in their L2. All the participants were students at Universität Göttingen and fluent in German; they had gone through German‐language secondary education (most also had German‐language primary education and kindergarten) and self‐reported German to be their L1 and dominant language. We asked participants about language use, such as when, with whom, and how often they used an L2. After an interview, we administered the KBIT‐2 test (participants had 15 min maximum to complete the test) and rapid picture‐naming test (first in German and then in L2). During a second session, the participants performed language tasks. For their time and effort, the participants received €20 compensation (data collection occurred in two acquisition periods: November 2022 and May 2023).

### 2.9 Participants

Our initial aim was to recruit German monolinguals, German–Turkish bilinguals, and German–Russian bilinguals, 35 participants per group, to achieve a significance level given a medium effect size in an omnibus ANOVA (with interactions), with alpha and beta thresholds set to 0.05 and 0.8, respectively (conventional values for social sciences); moreover, we expected large or medium effect sizes (*f* > 0.3) on the basis of previous studies [37, 66, 91]. However, our final sample groups slightly exceeded the initial desired size because, according to the procedure approved by the institutional ethical board, we had to run the experiment with all willing volunteers who subscribed before recruitment was stopped, and thus, 36 German–Turkish bilinguals and 41 monolingual German participants were recruited. Unfortunately, the available sample of German–Russian bilinguals was limited, and we, therefore, collected a mixed sample of 39 bilinguals whose additional language was Russian, Czech, or Russian. The utility of building a mixed group of German–Slavic bilinguals was based on the typological (structural) proximity of Slavic languages. Moreover, the typological distance between Turkish and German is greater than between German and any of the Slavic languages. So, the group of German–Slavic bilinguals allowed testing of our main hypothesis. All participants were students of the Universität Göttingen, providing homogeneous samples in terms of educational background and age and removing level of education, known to affect metacognition, as a confound.

In the German–Turkish bilingual sample, all participants but one were born in Germany (the exception came to Germany at 2 years of age) and acquired Turkish at home and in the community environment. Eighteen people reported to regularly follow religious practices and attend mosque; 11 participants reported not being religious. All Turkish bilinguals reported being literate in both languages and regularly read and wrote in their L2.

In the German–Slavic bilingual sample, 11 participants were born outside Germany, of whom 10 arrived in Germany between 2 and 10 years of age and went through German‐speaking secondary (and, if relevant, primary) education. Importantly, they had German as their heritage home language in their country of origin (hence, they were exposed to German from birth). One German–Slavic participant arrived at the age of 16. Although he had high proficiency in professional German (learning it via formal training), lexical access to common words (household objects, animals, and body parts) was more efficient in Russian than in German (in the rapid picture naming test, he named all 65 objects in Russian but only 58 objects in German—a pattern that is contrary to all other bilingual participants, who named 65 objects in German and 65 or fewer objects in their additional language); therefore, he did not meet the inclusion criteria and was excluded from the sample. Twenty‐eight German–Slavic bilinguals reported not being religious; 11 said that they attended the church regularly and followed religious practices. Six participants said that they were not literate in their L2 and four other participants (Russians) said that they could read but not write in Cyrillic script.

We subsequently screened the bilingual participants for their proficiency in L2 and compared their relative proficiency. First, we removed all participants who revealed large differences in relative proficiency (∆Prof > 20, meaning that a participant did not remember every third object in their additional language; we removed 1 German–Turkish and 3 German–Slavic bilinguals). As all other ∆Prof values were ≤10, the removed scores were considered outliers (defined as the values exceeding ±3 SD from the mean of the other scores). Afterward, we screened participants for IQ. On the basis of the IQ text, we removed one German–Slavic bilingual and one German monolingual because of IQ scores over three standard deviations lower than the mean score of other participants in respective samples.

## 3 Results

### 3.1 Relative Proficiency and IQ

**FIGURE 2:**
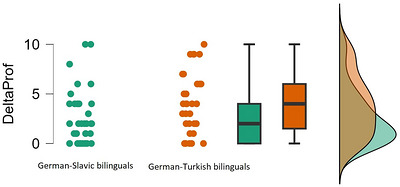
DeltaProf (∆Prof) in two bilingual samples. Smaller values show more balanced proficiency (smaller difference in proficiency in bilingual's languages is reflected in small ∆Prof value).

With outliers removed from the samples, we compared the relative proficiency between the samples via a Kruskal‒Wallis test (the test was chosen because ∆Prof is an ordinal variable and not normally distributed; most bilinguals are balanced; hence, the density is left‐skewed). The difference in ∆Prof was not significant, *H*(1) = 3.644, *p* = 0.056, η^2^ = 0.039 (Figure 2), which does not support the assumption that the relative proficiency between L1 and L2 differs between bilingual samples.

**FIGURE 3:**
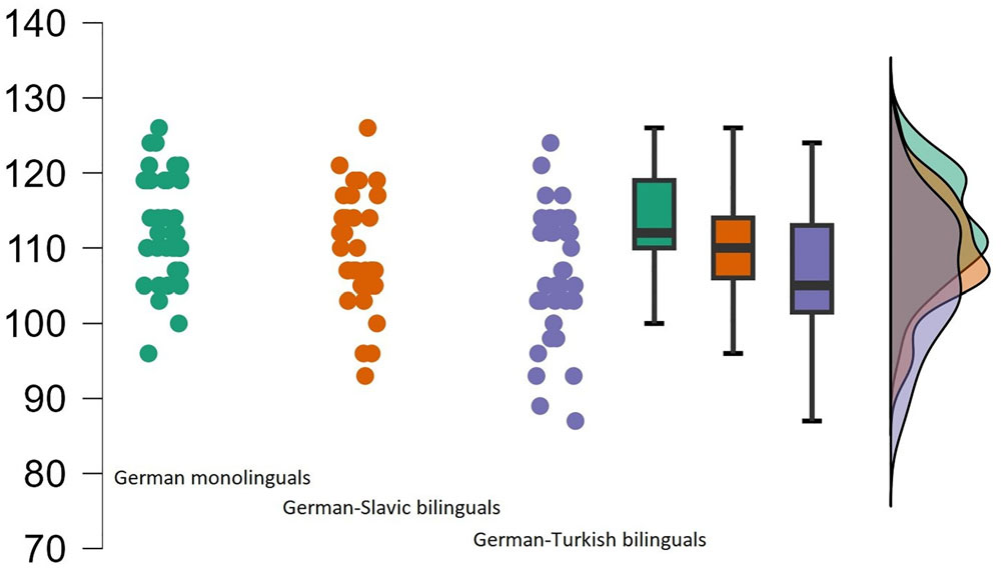
Individual scores on logical IQ (KBIT‐2, logical part, adjusted‐for‐age score) in three samples under investigation.

The logical IQ value, however, was significantly different between groups, *F*(2, 107) = 6.838, *p* = 0.002, $\eta_{p}^{2}$ = 0.113. Pairwise comparisons (with the Bonferroni‒Holm correction) revealed that the logical IQ is 0.86 standard deviations greater in the group of German monolinguals than in the group of Turkish‒German bilinguals, *t*(107) = 3.697, *p *< 0.001. Differences between German monolinguals and German–Slavic bilinguals (*p* = 0.136) and between German–Slavic bilinguals and German–Turkish bilinguals (*p* = 0.136) are not significant (Figure 3).

### 3.2 Statistical Learning

**FIGURE 4:**
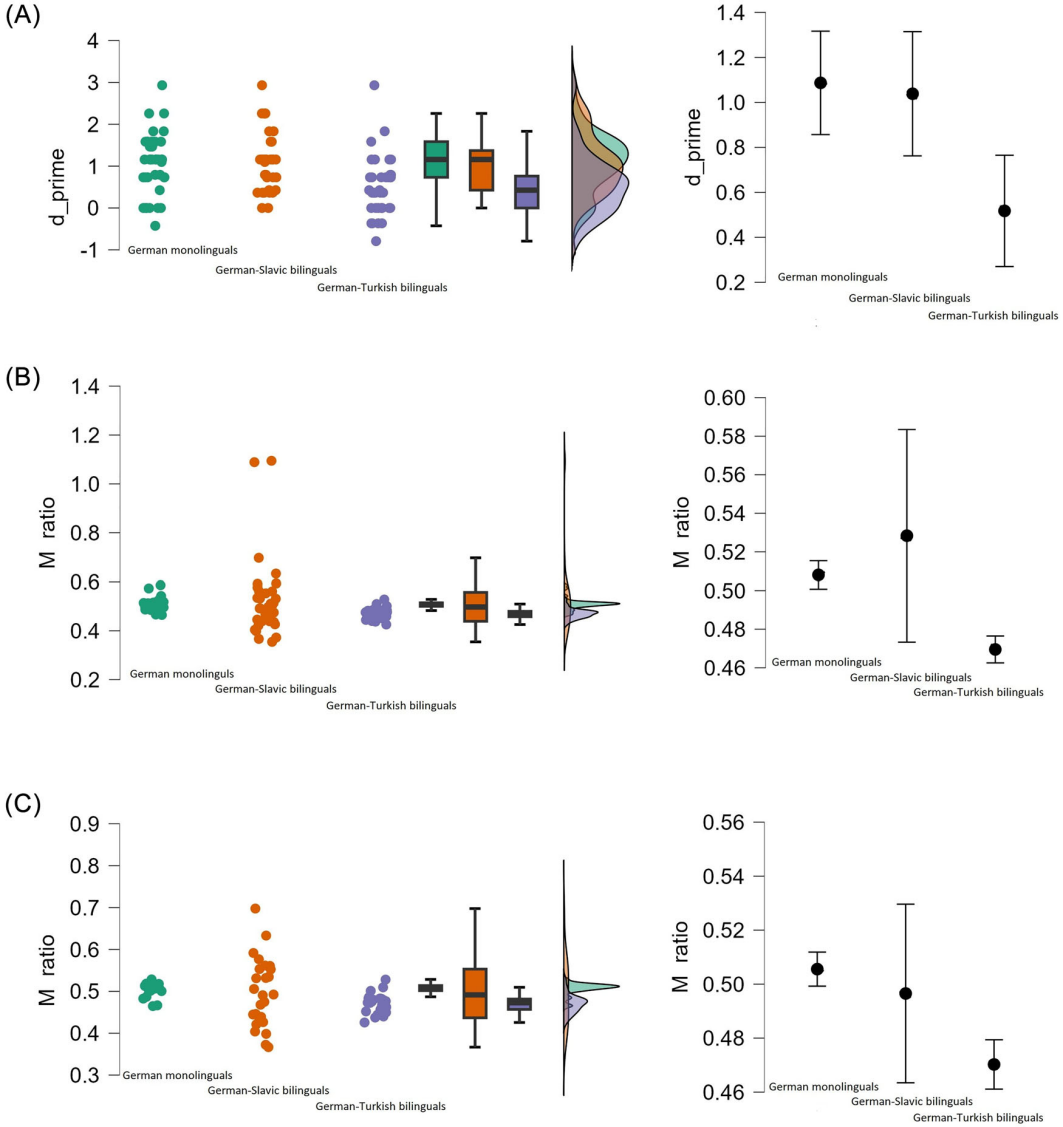
Statistical learning experiment: (A) Left: *d*′ individual datapoints, probability density, and boxplots (showing median and IQR). Right: *d*′ means, error bars represent 95% CI. (B) Left: *M*‐ratio individual datapoints, probability density, and boxplots (showing median and IQR). Right: *M*‐ratio means, error bars represent 95% CI. (C) Left: M‐ratio individual datapoints, probability density, and boxplots (showing median and IQR) on trimmed data (data trimmed for IQ, individuals with IQ exceeding 120 and below 100 were excluded from the sample, which led to insignificant differences in IQ between the groups; the analysis was performed on the trimmed dataset). Right: M‐ratio means on the trimmed dataset, error bars represent 95% CI.

Differences in *d*′ between the groups were significant, *F*(2, 107) = 6.42, *p* = 0.002, $\eta_{p}^{2}$ = 0.107. Pairwise comparisons revealed that performance was lower in the group of Turkish bilinguals than in the group of German–Slavic bilinguals, *t*(107) = 2.915, *p* = 0.009, and in the group of German monolinguals, *t*(107) = 3.29, *p* = 0.004 (all *p* values here and further adjusted for multiple comparisons via the Bonferroni‒Holm method). No significant difference was observed in *d*′ between monolingual German and bilingual German–Slavic populations, *p* = 0.781. This pattern can be observed in Figure 4A.

*M*‐ratio scores (the measure of metacognitive efficiency) were not normally distributed; hence, we used the Kruskal‒Wallis test to probe the differences in metacognition between groups. The test revealed a significant difference, *H*(2) = 24.5, *p* < 0.001, rank $\eta_{p}^{2}$ = 0.21. Dunn's pairwise comparisons revealed that meta‐efficiency in the group of German–Turkish bilinguals was lower than that in the group of German–Slavic bilinguals, *z* = 3.076, *p* = 0.004 (corrected), *r*_rb_ = 0.256, and in the group of German monolinguals, *z* = 4.909, *p* < 0.001 (corrected), *r*_rb_ = 0.809. No significant difference was observed in the *M*‐ratio between monolingual German and bilingual German–Slavic populations, *p* = 0.25 (see Figure 4B).

The correlations between metacognitive efficiency (*M*‐ratio) and ∆Prof were not significant (rho = −0.088, *p* = 0.469). Importantly, we observed a moderate yet significant correlation between IQ and the *M*‐ratio (rho = 0.268, *p* = 0.005, *z* = 0.274); hence, the observed differences in the *M*‐ratio between the groups could be driven by group‐level IQ differences between our samples. However, using IQ as a covariate in the analysis of variance would also regress out the effect of the group. To address this concern, we trimmed the data by removing individuals with high and low IQ values across all groups (below 100 and above 120). The trimmed subsamples included 25 German monolinguals, 27 German–Slavic bilinguals, and 26 German–Turkish bilinguals. ANOVA did not reveal differences in IQ in the trimmed sample, *F*(2,75) = 0.226, *p* = 0.798. This made it possible to use IQ as a covariate in the ANCOVA, which still revealed the effect of the group, *F*(2,73) = 3.188, *p* = 0.047, when controlling for the effect of IQ at the individual level (see Figure 4C), in the absence of group‐level differences in the IQ on the trimmed samples. The results of the trimmed sample recapitulated the results of the untrimmed sample.

### 3.3 Word Learning

**FIGURE 5:**
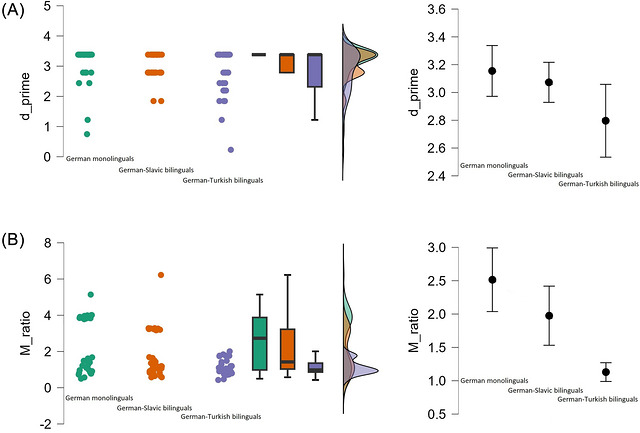
Inferential word learning (semantic task) experiment: (A) Left: *d*′ individual datapoints, probability density, and boxplots (showing median and IQR). Right: *d*′ means, error bars represent 95%CI. (B) Left: *M*‐ratio individual datapoints, probability density, and boxplots (showing median and IQR). Right: *M*‐ratio means, error bars represent 95%CI.

Differences in *d*′ between the groups in the inferential word learning (semantic task) experiment were significant, *H*(2) = 8.014, *p* = 0.018, $\eta_{p}^{2}$ = 0.056 (the nonparametric test was chosen because the assumption of normality and the assumption of homogeneity were violated). Pairwise comparisons revealed that performance was lower in the group of German–Turkish bilinguals than in the group of German monolinguals, *z* = 2.819, *p* = 0.014 (corrected). We did not observe significant differences in performance between German–Slavic bilinguals and German monolinguals, *p* = 0.365, or between German–Slavic and German–Turkish bilinguals, *p* = 0.218. This pattern can be observed in Figure 5A.

Metacognitive efficiency was significantly different between groups, *H*(2) = 14.85, *p* < 0.001, $\eta_{p}^{2}$ = 0.12, *r*_rb_ = 0.464. No significant difference was observed in the *M*‐ratio between monolingual German and bilingual German–Slavic populations, *p* = 0.321, or between German–Slavic and German–Turkish bilingual populations, *p* = 0.091 (corrected by Bonferroni). This pattern can be observed in Figure 5B.

The correlations between ∆Prof and the *M*‐ratio (rho = 0.134, *p* = 0.267) and between IQ and the *M*‐ratio (rho = 0.103, *p* = 0.292) in the semantic task were weak and not significant (probably because the task was too easy).

### 3.4 Artificial Grammar Learning

**FIGURE 6:**
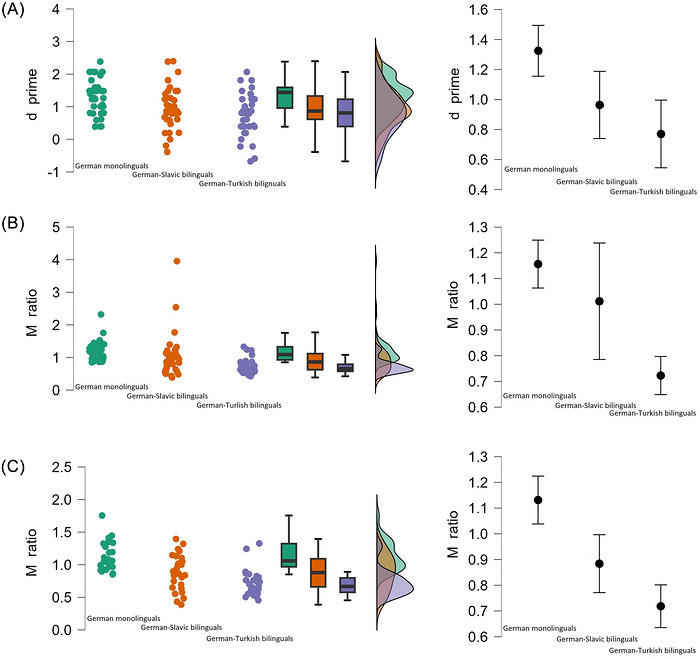
Artificial grammar learning experiment: (A) Left: *d*′ individual datapoints, probability density, and boxplots (showing median and IQR). Right: *d*′ means, error bars represent 95%CI. (B) Left: *M*‐ratio individual datapoints, probability density, and boxplots (showing median and IQR). Right: *M*‐ratio means, error bars represent 95%CI. (C) Left: *M*‐ratio individual datapoints probability density and boxplots (showing median and IQR) on trimmed data (data trimmed for IQ, individuals with IQ exceeding 120 and below 100 were excluded from the sample, which led to insignificant differences in IQ between the groups; the analysis was performed on the trimmed dataset). Right: *M*‐ratio means on the trimmed dataset, error bars represent 95%CI.

Differences in *d*′ in the artificial grammar learning (syntactic task) experiment between the groups were significant, *F*(2, 107) = 7.993, *p* < 0.001, $\eta_{p}^{2}$ = 0.13. Pairwise comparisons revealed that performance is 0.59 standard deviations lower in the group of German–Slavic bilinguals than in the group of German monolinguals, *t*(107) = 2.549, *p* = 0.024. The performance of the German–Turkish group was lower than that of the German monolingual group by 0.906 standard deviations, *t*(107) = 3.914, *p* < 0.001. No significant differences in performance were observed between German–Slavic bilinguals and German–Turkish bilinguals, *t*(107) = 1.322, *p* = 0.189. All *p* values were adjusted for multiple comparisons via the Bonferroni‒Holm method. The reported pattern of results is displayed in Figure 6A.

In terms of metacognitive efficiency, we observed significant differences in the *M*‐ratio between groups, *H*(2) = 37.01, *p* < 0.001, $\eta_{p}^{2}$ = 0.327 (very substantial effect size). Dunn's pairwise comparisons revealed that all the pairwise differences between all the groups were significant. Meta‐efficiency was greater in the group of monolingual German speakers than in the group of German–Turkish bilinguals, *z* = 6.052, *p* < 0.001, *r*_rb_ = 0.843, and in the group of German–Slavic bilinguals, *z* = 3.377, *p* = 0.001, *r*_rb_ = 0.423. The *M*‐ratio was greater in the group of German–Slavic bilinguals than in the group of German–Turkish bilinguals, *z* = 0.589, *p* = 0.01, *r*_rb_ = 0.324. The reported pattern of results is displayed in Figure 6B.

The correlations between metacognitive efficiency (*M*‐ratio) and ∆Prof were not significant (rho = 0.086, *p* = 0.48). Importantly, we observed a moderate yet significant correlation between IQ and the *M*‐ratio (rho = 0.259, *p* = 0.007, *z* = 0.265). To confirm the result pattern by controlling for IQ, we ran ANCOVA on the trimmed sample (removing individuals with extremely high and low IQ values made the differences in IQ between samples insignificant, see the analysis of the statistical learning experiment) and introduced IQ as a covariate. When controlling for individual IQ, the effect of group was still significant, *F*(2,74) = 18.84, *p* < 0.001, $\eta_{p}^{2}$ = 0.34. The effect of IQ was not significant (*p* = 0.277). Pairwise differences also revealed that all pairwise differences in the *M*‐ratio of the trimmed sample were significant, which perfectly recapitulates the pattern observed in the untrimmed data (Figure 6C).

### 3.5 Analysis of Global Metacognition

Global metacognition is related to individuals’ self‐assessment of how well they are doing the task overall, and it might differ from local metacognition, manifested in trial‐by‐trial adjustments of confidence ratings. The number of data points of local metacognition per participant in this study is equal to the number of trials, whereas we can only have one data point of global metacognition per participant per task.

We collected two estimates per participant at the end of the experiment: How well one believes (s)he has done the task (binary response) and how sure one is in this response (four‐point scale). We mapped these answers onto one scale from one to eight, according to Table 1. After that, we calculated Spearman correlations for each task for each group (Table 2) and compared these correlations statistically (Table 3).

**TABLE 1:** Mapping of responses to two final questions after the test to the global self‐evaluation scale.

How well do you think you did the task	How sure are you in this self‐assessment	The resulting score (combined score)
Bad	Totally sure	1
Bad	Sure	2
Bad	Somewhat sure	3
Bad	Not sure	4
Well	Not sure	5
Well	Somewhat sure	6
Well	Sure	7
Well	Totally sure	8

**TABLE 2:** Correlations between *d*′ (cognitive performance of an individual in a particular task) and the self‐estimation of one's own performance in this task (combined score).

	Word learning	Statistical learning	Artificial grammar learning
Monolingual Germans (*N* = 40)	Rho = 0.33, *p* = 0.038	Rho = 0.068, *p* = 0.678	Rho = 0.128, *p* = 0.431
German–Turkish bilinguals (*N* = 35)	Rho = 0.297, *p* = 0.083	Rho = 0.373, *p* = 0.027	Rho = 0.508, *p* = 0.002
German–Slavic bilinguals (*N* = 35)	Rho = 0.559, *p* < 0.001	Rho = 0.316, *p* = 0.064	Rho = 0.071, *p* = 0.684
Interpretation	Global metacognition in the group of German–Slavic bilinguals is higher than in the other two groups	Global metacognition in the group of German monolinguals is lower than in the other two groups	Global metacognition in the group of German–Turkish bilinguals is higher than in the other two groups

*Note*: A higher correlation indicates greater global metacognition in a particular group on a particular task.

**TABLE 3:** A comparison of the correlation coefficients is reported in Table 2.

	Word learning	Statistical learning	Artificial grammar learning
**German monolinguals vs. German–Turkish bilinguals**	n/s	n/s	German monolinguals < German–Turkish bilinguals, *z* = 2.173, *p* = 0.016
**German monolinguals vs. German–Slavic bilinguals**	n/s	n/s	n/s
**German–Slavic bilinguals vs. German–Turkish bilinguals**	n/s	n/s	German–Slavic < German–Turkish bilinguals, *z* = 2.294, *p* = 0.011
**Interpretation**	Overall, global metacognition is lower in the group of German monolinguals across all tasks, although the only difference that survives significance testing is in the artificial grammar learning task: on this (syntactic) task, global metacognition in the groups of German–Turkish bilinguals compared to German–Slavic bilinguals and German monolinguals		

## 4 Discussion

The data showed that heritage languages with limited social use provide no local metacognitive advantage, whereas global metacognition may still be enhanced in bilinguals speaking a heritage language. Local metacognition in the group of German–Turkish bilinguals was lower than that in the other groups (German–Slavic bilinguals and German monolinguals) across all three language tasks (semantic, syntactic, and speech segmentation tasks). We did not observe a significant difference in metacognition between German–Slavic bilinguals and German monolinguals in speech segmentation and semantic tasks, but in syntactic tasks—artificial grammar learning—metacognition was significantly greater in the group of German monolinguals. This pattern remained stable after controlling for other potential factors, including IQ, demographic factors, and relative proficiency in bilinguals’ languages. Global metacognition at the group level, on the other hand, was lower across all tasks in the group of German monolinguals.

The observed result pattern in relation to local metacognitive performance is contrary to predictions and to the previously reported bilingual advantage in metacognitive processing on language tasks. Ordin et al. [37] and Polyanskaya et al. [41] reported that Basque–Spanish bilinguals exhibited substantially and significantly superior metacognitive processing than Spanish monolinguals did, with Catalan–Spanish bilinguals showing intermediate metacognitive performance. The pattern we observed in bilingual populations in Germany is the opposite: Bilinguals speaking typologically different languages (Turkish and German) exhibit inferior local metacognition compared to monolingual speakers. Hence, we confirmed neither bilingual advantage nor the advantage elicited by typological, structural differences between bilinguals’ languages. This echoes the conclusion of recent sociolinguistic studies that multilingual speakers often exhibit cognitive patterns unpredictable by typological distance of languages in bilinguals’ inventory, or by monolingual‐bilingual contrasts more generally [92, 93, 94].

Notably, Slavic and Turkish languages are heritage languages in Germany, spoken mostly in the family environment or in close social circles (among friends or in neighborhoods, ethnic communities, at the mosque), whereas German is used as an official language in the administrative domain, professional communication, and educational settings (there are quite a few Russian bilingual schools in Germany, but we did not recruit people who attended bilingual schools and included only participants with a Slavic language—Russian, Polish, or Czech—as a heritage home or community language). This is a major difference from the Basque‐Spanish and Catalan–Spanish populations in Spain, where local languages (Basque and Catalan) enjoy the status of official languages used at governmental establishments as administrative languages. Moreover, in respective administrative units (*communidades autonomas*—Catalunya and Pais Vasco), proficiency in a local language is a legal prerequisite for having a job as a public servant in many governmental offices or sectors—the state health system, public administration, and educational establishments. It is also expected that any visitor can be attended to in any official language (s)he prefers, for example, Spanish or Catalan/Basque. Additionally, local languages are used as a medium of education at schools and universities (not as a subject but as a language of instruction).

Ordin and Polyanskaya [66] demonstrated that using a second or minority language may be an influential factor that modulates metacognitive advantage in the language domain. They measured metacognition in syntactic, semantic, and speech segmentation tasks in Danish–German bilinguals residing in and attending educational institutions in Denmark and Germany, who either use or do not use the minority language as a medium of education. As the bilinguals in that study had the same set of languages in their inventory, the effect of linguistic structures was not a part of the design. The bilinguals were also matched in IQ and relative proficiency in both languages. The results showed that those bilinguals who had to resort to a minority language (Danish in Germany and German in Denmark) as a medium of education exhibited enhanced metacognition in language domains. This suggests that the effect observed in bilingual populations in Spain can also be attributed to the linguistic situation, which is very different from what is observed in Turkish and Russian communities in Germany, where Slavic and Turkic languages are used as heritage rather than as official languages. The results of the current study suggest that sociolinguistic factors related to language use and prestige can override language‐internal factors (structural properties of bilingual's languages) as driving forces of metacognitive efficiency.

Potentially, differences in metacognition between German–Turkish and German–Slavic bilinguals can be accounted for by institutional support of the heritage languages [94, 95]. Both Russian and Turkish are among the most widely researched heritage languages in Germany; however, they have different sociolinguistic positions. Turkish is rooted in a longer, multi‐generational migration history related to labor migration since the 1960s. At the same time, it remains associated with informal peer‐group and community domains. It is primarily used as a means of communication within ethnicity‐based communities [94]. Russian, by contrast, is linked largely to post‐Soviet migration (2 generations on average) and largely serves as a means of cultural identification [96]. Turkish is maintained through contact‐rich bilingual interactions involving code‐switching and mixed styles, whereas Russian may experience reduced exposure outside the family, and richer input is received via formal educational settings rather than through naturalistic interactions [97]. These differences might have implications for cognitive outcomes, including metacognitive processes.

It was most intriguing to observe that the hypothesis was confirmed for global metacognition (bilingual speakers with typologically different languages, German–Turkish bilinguals, exhibit more acute global metacognitive sensitivity than monolingual German speakers do). Global metacognition is not linked to individual decisions but rather to the gradual formation of self‐performance estimates in a particular task, considering one's own self‐assessment in the task per se, in a specific cognitive domain, and in one's own cognitive capabilities in general [15, 75]. It might be surprising to observe different patterns of global and local metacognition, especially considering that they rely on partially overlapping neural networks [98], and functionally, metacognitive trial‐by‐trial (or decision‐by‐decision) sensitivity serves to build self‐confidence in the task overall [14, 15]. However, global and local metacognition are differentially affected by neurodegeneration changes (e.g., in patients with cognitive disorders, including Alzheimer's disease, global metacognition is affected, whereas local metacognition is preserved) [75, 99]. With age, deterioration in global metacognition is observed despite intact local metacognitive monitoring efficiency [13]. Moreover, the accumulation of evidence needed to evaluate the general ability to perform a task is mostly an unconscious process, whereas the methods most widely used to assess local metacognition are based on retrospective confidence ratings, when a participant is asked to deliberately and consciously evaluate one's own recently made decision and assess the confidence rating of this decision, which increases the contribution of conscious awareness in self‐evaluations. Conscious and unconscious awareness of one's own performance differentially contributes to metacognition [100, 101], suggesting a possible differentiation of global and local metacognitive processes due to differences in the ratio of conscious to unconscious awareness. Therefore, theoretically, it is possible to observe different results with respect to local versus global metacognitive efficiency, which is also confirmed by our empirical data.

Importantly, global and local metacognition have distinct functional roles in the regulation of behavior. Local metacognition allows flexibility within a particular task or domain, adapting decision‐making strategies to a changing environment. The goal is to increase the efficiency of the task at hand. Global metacognition, on the other hand, affects future decisions on a longer time scale, influences self‐esteem, determines recovery time after failure, and impacts the likelihood of making life‐changing decisions [15, 27]. The goal is to choose the task or domain in which one can be more efficient. If language experience (bilingual experience, language use, educational media, etc.) may affect global and local metacognition differentially, it may account for different decisions made by the same people in equivalent situations. The reliance on local versus global metacognition in decision‐making may influence the perception of one's own and others’ activities in relation to individual success. In the context of environmental uncertainty, enhanced global metacognition might encourage people to select the domain in which they are more confident and more success prone, and enhanced local metacognition might encourage people to adapt behavioral strategies to remain successful in the chosen field of activities. This hypothesis, however, requires further testing against real‐world data.

We acknowledge a limitation of the study, especially with regard to extralinguistic factors that might influence metacognition. Some recent evidence—largely correlational rather than causal—suggests that socioeconomic factors might be related to metacognition [102, 103]. We do not have information about differences in the socioeconomic status of our participants or whether socioeconomic status differs between Slavic–German and Turkish–German bilinguals (we were not allowed to collect this information). Socioeconomic factors may potentially influence metacognition because metacognitive knowledge, monitoring, and regulation develop within learning environments shaped by parental education, income, language input, and cognitively stimulating interactions. The effect of socioeconomic status on metacognition will remain an open question for further investigations.

## 5 Conclusions

We conclude that sociolinguistic factors might override the effect of purely language experience on metacognitive efficiency. A bilingual advantage in metacognition can be observed when both languages in bilinguals’ inventories are used across all operational domains, not kept apart different situational contexts (hence, the heritage language that is used in a limited set of social contexts does not have a beneficial effect on local metacognition). The most important operational domain is the use of language as a medium of education. However, global metacognition (which can be modulated differentially from local metacognition or adjusting confidence decision‐by‐decision) is enhanced in bilingual populations even if the additional language is a heritage language with a limited range of use.

## Author Contributions

Both authors equally contributed to conceptualization of the study, experiments development, data analysis, and writing up.

## Conflicts of Interest

The authors declare no conflicts of interest.

## Data Availability

The data are available from the corresponding author—upon anonymization—at any time.
